# Cognitive function and quality of life in multiple sclerosis patients: a cross-sectional study

**DOI:** 10.1186/1471-2377-11-17

**Published:** 2011-02-02

**Authors:** Karine Baumstarck-Barrau, Marie-Claude Simeoni, Françoise Reuter, Irina Klemina, Valérie Aghababian, Jean Pelletier, Pascal Auquier

**Affiliations:** 1 EA3279 Self-perceived Health Assessment Research Unit and Department of Public Health, Nord University Hospital, APHM, Marseille, France; 2 Departments of Neurology and CRMBM CNRS6612, Timone University Hospital, APHM, Marseille, France; 3 EA 3273 Psychology of Cognition, Language, and Emotion Research Centre, Aix-Marseille University, France

## Abstract

**Background:**

Nearly half of all patients diagnosed with multiple sclerosis (MS) will develop cognitive dysfunction. Studies highlighted from no/weak impact to a strong impact of cognitive impairment on quality of life (QoL). The aim of this study was to assess the impact of cognitive dysfunction on self-reported QoL in MS patients while considering key confounding factors.

**Methods:**

Design: cross-sectional study. Inclusion criteria: MS patients of any disease subtype. Data collection: sociodemographic (age, gender, marital status, education level, and occupational activity) and clinical data (MS subtype, disease duration); MS disability (Expanded Disability Status Scale, EDSS); depression (Beck Depression Inventory); fatigue (Modified Fatigue Impact Scale); QoL (SF36 and MusiQoL); and neuropsychological performance (Brief Repeatable Battery of Neuropsychological Tests, BRB-N). Statistical analysis: multiple linear regressions (forward-stepwise selection).

**Results:**

One hundred and twenty-four patients were enrolled. Performance on BRB-N subtests varied widely (6% to 70% abnormal). The BRB-N classified 37-78% of the patients as cognitively impaired, depending on the definition of cognitive impairment. No links were found between the MusiQoL index and cognitive subtests, whereas marital status, EDSS, and depression were found to be independent predictive factors.

**Conclusions:**

The present study demonstrated the weak and scarce association between cognitive impairment and QoL, when the key confounding factors were considered. These results need to be confirmed with larger samples and more accurate tests of cognitive function.

## Background

Multiple sclerosis (MS) is the most common demyelinating disease of the central nervous system in young adults. Cognitive impairment occurs in about 50% of patients with MS [1,2], even during the early stages of the disease [3,4]. Cognitive dysfunction may subsequently result in reduced fulfilment in work life and social life as well as in a reduction in quality of life (QoL) [5-7]. To date, few studies have reported relationships between cognitive function and patient QoL [8,9]. Existing studies have revealed contradictory results, highlighting either negligible impact [7,10-13] or a strong impact [5,14,15] of cognitive impairment on QoL. The potential weaknesses of these studies could lie in the cognitive or QoL evaluations. In some studies, the cognitive assessment was restricted to a single cognitive function [14,15]. In others, cognitive impairment was defined using tools recognised as insufficiently sensitive [5,12]. In still other studies, the QoL assessment was restricted to a single QoL-specific domain [11], or QoL predictors were not considered simultaneously [12,14]. Thus, it was necessary to assess the relationship between cognitive performance and QoL using a standardised neuropsychological battery and a disease-specific patient-based instrument, respectively. To our knowledge, only one previous study has reported this relationship using both a well-established battery of cognitive tests (Brief Repeatable Battery of Neuropsychological Tests) and a well-validated QoL measure (Multiple Sclerosis Quality of Life Inventory) [16]. However, this study focused on early MS, and depression was the only controlled variable. We examine the relationship between cognitive dysfunction and QoL in a sample of patients with MS, including any disease subtypes, while considering the key sociodemographic and clinical confounding factors to report that cognitively disabled patients with MS are able to give consistent answers to self-reported questionnaires.

## Methods

This study incorporated a cross-sectional design and was performed in the neurology department of a French public academic teaching hospital (Marseille, France). The inclusion criteria were as follows: MS patients according to McDonald criteria [17,18], any disease subtype, no history of psychiatric or neurological disease (other than MS), no history of alcohol/drug abuse, and native French speakers. The French Ethics Committee (Comité de Protection des Personnes, Marseille II) approved the study, and patients gave their informed consent to participate. Sociodemographic (age, gender, marital status, education level, and occupational activity) and clinical (MS subtype, disease duration) data were recorded. The MS disability was assessed using the Expanded Disability Status Scale (EDSS). Depression was assessed using the self-administered Beck Depression Inventory (BDI) [19], and fatigue was assessed using the 21-item Modified Fatigue Impact Scale (MFIS) [20].

Two QoL questionnaires were administered. The Short Form 36 (SF36) is a generic questionnaire [21] describing eight subscales (physical function, social functioning, role physical, role emotional, mental health, vitality, bodily pain, and general health). Two composite scores (physical and mental, PCS-SF36 and MCS-SF36) can also be calculated. The Multiple Sclerosis International Quality of Life (MusiQoL) is a well-validated, disease-specific questionnaire [22] describing nine dimensions (activity of daily living (ADL), psychological well-being (PWB), symptoms (SPT), relationships with friends (RFr), relationships with family (RFa), relationships with health care system (RHCS), sentimental and sexual life (SSL), coping (COP), and rejection (REJ)) and yielding a global index score.

Neuropsychological performance was assessed using the Brief Repeatable Battery of Neuropsychological Tests (BRB-N) [23]. The eight subtests were selected to explore most of the cognitive functions: the three indices of the Selective Reminding Test (SRT-L, SRT-C, SRT-D), a verbal learning/memory test; the two indices of the 10/36 Spatial Recall Test (SPART-T, SPART-D), a visuospatial learning and memory test; the Symbol Digit Modalities Test (SDMT), which determines the speed of visual information processing, complex visual scanning, and sustained attention; the Paced Auditory Serial Addition Test (PASAT-3), involving mental calculation, working memory, concentration, and information processing speed; and the Word List Generation test (WLG), a semantic verbal fluency test. The battery was administered in a standardised way by the same psychologist (FR), who was intensively trained in test administration. Cognitive impairment or deficit was defined using Camp's normative values [24]. The subject was considered cognitively impaired or deficient for one subtest if the score was at least two SDs below the mean normative values, and he/she was considered cognitively impaired or deficient for the global battery if he/she was cognitively impaired for at least three of the eight subtests.

### Statistical analyses

Data were expressed as the means/standard deviations or the medians/ranges, depending on the parametric or non-parametric distribution of the variable. Associations between the QoL scores and continuous variables (fatigue, depression, BRB-N subtests, age, EDSS, disease duration) were analysed using Spearman's correlation tests. Means-based comparisons of the MusiQoL dimensions and the indices between different sub-groups (gender, educational level, marital status, living, occupational status, MS subtype, BRB-N cognitive function) were calculated using Mann-Whitney or Kruskal-Wallis tests. To determine variables potentially predictive QoL levels, multiple linear regressions (forward-stepwise selection) were performed. The MusiQoL index and each of the MusiQoL dimensions were considered as separate dependent variables. The variables relevant to the models were selected from the univariate MusiQoL index analysis, based on a threshold p-value ≤0.20 (MFIS-To, BDI) or a threshold p-value ≤0.30 for the BRB-N subtests. A set of additional variables was included in the models owing to their clinical and sociodemographic relevance (gender, age, marital status, EDSS, MS subtype, and disease duration). The final models incorporated the standardised beta coefficients. The independent variables with the higher standardised beta coefficients are those with a greater relative effect on QoL. The statistical analyses were performed using the SPSS software package version 15.0 (SPSS Inc., Chicago, IL, USA). All tests were two-sided. Statistical significance was defined as p < 0.05.

## Results

One hundred and twenty-four consecutive inpatients and outpatients with MS were enrolled during a twelve-month period. The mean age was 45.05 years (SD 10.80), the sex-ratio was 0.75, 47.2% were single, 47.2% had more than 12 years of education, 64.5% were unemployed, and 87.3% were living in a personal home. The MS subtypes included 61 secondary progressive, 36 relapsing remitting, 20 primary progressive, and 7 clinically isolated syndromes. Performance on BRB-N subtests varied widely (6% to 70% abnormal). The BRB-N classified 37-78% of the patients as cognitively impaired, depending on the definition of cognitive impairment. The clinical features, self-reported data, and cognitive data are listed in table 1.

**Table 1:** Clinical characteristics, self-reported quality of life and cognitive functions

		N = 124
Disease duration in years	median [range]	9.86 [0-31.00]

EDSS^a^	median [range]	4.75 [1.00-8.00]

Depression	BDI^b ^(0-39)	10.42 ± 5.77
	BDI^b ^classes (moderate/severe depression)	47 (70.1%)

Fatigue	MFIS^c ^Physical (0-36)	25.97 ± 7.27
	MFIS^c ^Cognitive (0-40)	18.75 ± 8.87
	MFIS^c ^Psychosocial (0-8)	5.35 ± 2.07
	MFIS^c ^Total (0-84)	50.03 ± 15.31

SF36^d^	Physical function (0-100)	33.88 ± 26.78
	Social functioning (0-100)	50.88 ± 23.09
	Role physical (0-100)	20.95 ± 30.62
	Role emotional (0-100)	32.28 ± 38.16
	Mental health (0-100)	49.90 ± 17.81
	Vitality (0-100)	32.48 ± 19.12
	Body pain (0-100)	45.80 ± 26.21
	General health (0-100)	39.72 ± 20.28
	Mental composite score	32.64 ± 8.83
	Physical composite score	38.59 ± 9.53

MusiQoL^e^	Activity of daily living (0-100)	29.94 ± 21.24
	Psychological well-being (0-100)	50.97 ± 24.79
	Relationships with friends (0-100)	62.03 ± 24.21
	Symptoms (0-100)	54.31 ± 23.85
	Relationships with family (0-100)	72.17 ± 24.89
	Relationships with health care system (0-100)	69.49 ± 19.48
	Sentimental and sexual life (0-100)	49.19 ± 31.75
	Coping (0-100)	55.64 ± 29.40
	Rejection (0-100)	66.00 ± 33.42
	Index (0-100)	56.63 ± 12.57

BRB-N subtests^f^	SRT-L^g^	46.44 ± 15.27
	SRT-C^g^	40.17 ± 15.72
	SRT-D^g^	9.37 ± 2.96
	SPART-T^g^	16.40 ± 5.20
	SPART-D^g^	5.29 ± 2.36
	SDMT^g^	35.68 ± 11.92
	PASAT-3^g^	34.15 ± 15.02
	WLG^g^	25.32 ± 8.40

^a^EDSS Expanded Disability Status Scale; the higher the score, the more severe the disability

^b^BDI Beck Depression Inventory; the higher the score, the worse the depression

^c^MFIS Modified Fatigue Impact Scale; the higher the score, the more severe the fatigue

^d^SF36 Short Form 36; the higher the score, the higher the QoL level

^e^MusiQoL Multiple Sclerosis International Quality of Life; the higher the score, the higher the QoL level

^f^BRB-N subtests; the higher the score, the higher the cognitive performance

^g^SRT-L Selective Reminding Test Long-term, SRT-C Selective Reminding Test Consistent long-term, SRT-D Selective Reminding Test Delayed, SPART-T SPAtial Recall Test Total, SPART-D SPAtial Recall Test Delayed, SDMT Symbol Digit Modalities Test, PASAT-3 Paced Auditory Serial Addition Test 3 s, WLG Word List Generation test

The QoL was not strongly associated with any cognitive subtests. The MusiQoL index was not significantly correlated with the BRB-N subtests. Weakly significant correlations were identified for the ADL, SPT, RFa, and SSL dimensions, indicating a higher cognitive performance given a higher QoL level, and for the REJ dimension, indicating a higher cognitive performance given a lower QoL level. Depression and fatigue (with the exception of the physical component) were significantly linked to the MusiQoL index and the two composite scores of the SF36. All correlations are presented in table 2.

**Table 2:** Correlations between QoL (MusiQoL and SF36) and the BRB-N subtests, fatigue, and depression

	MusiQoL ^a^										SF36^**b**^	
	ADL^**a**^	PWB^**a**^	RFr^**a**^	SPT^**a**^	RFa^**a**^	RHCS^**a**^	SSL^**a**^	COP^**a**^	REJ^**a**^	Index^**a**^	PCS^**b**^	MCS^**b**^
SRT-L^**c**^	-0.04	-0.02	-0.15*	0.17*	-0.08	-0.07	0.06	0.02	-0.22**	-0.14*	-0.07	-0.03
SRT-C^**c**^	-0.05	-0.08	-0.05	0.13*	-0.04	-0.04	0.11	-0.03	-0.25**	-0.08	-0.11	0.01
SRT-D^**c**^	0.02	-0.15*	-0.06	0.07	-0.02	-0.08	0.13*	0.02	-0.35***	-0.13*	-0.007	-0.03
SPART-T^**c**^	-0.06	-0.01	0.12*	0.06	0.21**	0.08	0.33***	0.18*	-0.05	0.16*	-0.19*	0.05
SPART-D^**c**^	-0.08	0.04	0.08	-0.06	0.22**	-0.03	0.24**	0.21*	-0.13*	0.06	-0.28***	0.13*
SDMT^**c**^	0.23**	0.01	-0.001	0.15*	0.05	-0.09	0.19*	0.16*	-0.20*	0.07	0.07	0.11
PASAT-3^**c**^	0.09	0.02	0.07	0.20**	0.06	0.09	0.16*	0.11	-0.26**	0.11	-0.003	0.23**
WLG^**c**^	0.12*	0.11*	0.09	0.24**	0.02	-0.11*	0.16*	0.15*	0.001	0.17*	0.03	0.17*

MFIS-Ph^**d**^	-0.58***	-0.17*	0.22**	-0.29***	0.20**	-0.08	0.02	-0.06	-0.21**	-0.11	-0.61***	-0.25**
MFIS-Cg^**d**^	-0.29***	-0.28***	0.05	-0.62***	-0.11*	0.05	-0.14*	-0.12*	-0.13*	-0.35***	-0.17*	-0.47***
MFIS-Ps^**d**^	-0.57***	-0.21**	0.02	-0.26***	0.14*	-0.24**	-0.08	-0.27***	-0.28***	-0.40***	-0.52***	-0.34***
MFIS-To^**d**^	-0.55***	-0.30***	0.11*	-0.52***	0.05	-0.07	-0.10	-0.17*	-0.23**	-0.34***	-0.47***	-0.44***

BDI^**e**^	-0.44***	-0.44***	-0.17*	-0.23*	-0.25**	-0.18*	-0.25*	-0.27**	-0.18*	-0.53***	-0.32**	-0.56***

^a^MusiQoL Multiple Sclerosis International Quality of Life, ADL activity of daily living, PWB psychological well-being, RFr relationships with friends, SPT symptoms, RFa relationships with family, RHCS relationships with health care system, SSL sentimental and sexual life, COP coping, REJ rejection, Index global score

^b^SF36 Short Form 36, PCS physical composite score, MCS mental composite score

^c^SRT-L Selective Reminding Test Long-term, SRT-C Selective Reminding Test Consistent long-term, SRT-D Selective Reminding Test Delayed, SPART-T SPAtial Recall Test Total, SPART-D SPAtial Recall Test Delayed, SDMT Symbol Digit Modalities Test, PASAT-3 Paced Auditory Serial Addition Test 3 s, WLG Word List Generation test

^d^MFIS-Ph Modified Fatigue Impact Scale physical, MFIS-Cg cognitive, MFIS-Ps psychosocial, MFIS-To total

^e^BDI Beck Depression Inventory

Spearman rank correlation coefficients were presented

*p-value < 0.30, **p-value < 0.05, ***p-value < 0.01

The MusiQoL index was not statistically linked to the sociodemographic/clinical status, except in the case of marital status; single patients had lower QoL scores. Relationships between the MusiQoL dimensions and sociodemographic/clinical variables are detailed in table 3. The variables selected for the multivariate models included gender, marital status, age, EDSS, MS subtype, disease duration, MFIS-To, BDI, and the four subtests with p-value ≤0.30 (SRT-L, SRT-D, SPART-T, WLG). No associations were found between the MusiQoL index and the cognitive subtests. Marital status, EDSS, and depression were associated with the MusiQoL index. No links between the MusiQoL dimensions and cognitive functions were identified, except in the case of SPART-T with RHCS. The QoL dimensions describing physical components, such as ADL and SPT, were related to depression and fatigue, respectively. The dimensions describing relationship aspects, such as RFr and RFa, were linked to gender and marital status, respectively. These results are summarised in table 4.

**Table 3:** Associations between MusiQoL dimension scores and sociodemographics, clinical characteristics, and cognitive function

	ADL^a^	PWB^a^	RFr^a^	SPT^a^	RFa^a^	RHCS^a^	SSL^a^	COP^a^	REJ^a^	Index^a^
Gender*										

Women	28.64 (21.36)	44.43 (26.21)	63.47 (24.36)	50.64 (24.70)	71.47 (26.54)	70.51 (18.23)	53.13 (31.49)	53.07 (29.99)	60.28 (34.96)	54.63 (11.88)
Men	31.72 (21.17)	59.68 (19.86)	60.20 (24.17)	59.29 (21.93)	73.14 (22.65)	68.14 (21.15)	44.21 (31.76)	59.30 (28.49)	73.89 (29.77)	59.28 (13.14)
p*	0.28	**0.002**	0.41	**0.04**	0.99	0.70	0.19	0.28	**0.03**	0.08

Educational level*										

<12 years	27.93 (16.61)	48.78 (25.54)	62.08 (25.51)	49.90 (24.71)	71.17 (27.02)	73.84 (18.91)	47.84 (33.64)	53.29 (28.59)	64.73 (34.30)	55.76 (13.00)
≥ 12 years	32.25 (25.54)	53.49 (23.87)	61.98 (22.80)	59.49 (21.91)	73.37 (22.28)	64.30 (19.04)	50.91 (29.50)	58.51 (30.41)	67.40 (32.70)	57.78 (12.09)
p*	0.79	0.36	0.81	**0.03**	0.91	**0.007**	0.71	0.36	0.77	0.31

Marital status*										

Single	31.04 (22.14)	50.00 (26.69)	56.25 (27.59)	51.73 (23.33)	63.30 (27.61)	65.30 (19.73)	34.89 (33.79)	52.88 (32.24)	65.50 (35.01)	52.53(14.44)
Married/partnership	28.94 (20.54)	51.81 (23.20)	66.73 (20.12)	56.60 (24.27)	79.86 (19.41)	73.13 (18.67)	59.09 (26.29)	58.41 (26.28)	69.32 (31.81)	59.57 (10.23)
p*	0.65	0.52	0.06	0.26	**0.002**	**0.05**	**0.001**	0.45	0.34	**0.03**

Living*										

Personal home	30.65 (21.96)	52.97 (24.22)	63.32 (23.41)	54.89 (24.25)	71.76 (25.04)	70.03 (19.48)	49.38 (31.92)	58.52 (28.65)	68.27 (32.93)	57.33 (12.47)
Friend/family home	27.91 (15.04)	35.90 (24.39)	54.55 (30.36	48.88 (19.84)	69.23 (27.08)	65.38 (20.08)	28.57 (25.73)	41.34 (32.03)	58.65 (32.02)	51.46 (14.00)
p*	0.88	**0.03**	0.22	0.36	0.79	0.35	0.10	0.07	0.23	0.31

Occupational status*										

Working	40.84 (22.53)	54.38 (26.65)	59.48 (23.12)	56.82 (24.19)	76.58 (25.35)	70.68 (20.33)	52.00 (30.34)	57.41 (27.13)	65.95 (31.32)	57.08 (14.44)
Not working	25.96 (19.20)	50.47 (23.83)	64.47 (24.35)	53.15 (24.19)	70.36 (25.01)	68.91 (19.43)	48.05 (33.13)	55.07 (30.61)	66.83 (33.80)	56.89 (11.95)
p*	**0.001**	0.57	0.20	0.52	0.20	0.58	0.62	0.76	0.80	0.80

MS subtype**										

RR^b^	39.33 (23.67)	48.61 (24.25)	59.31 (26.61)	48.11 (25.29)	65.53 (27.74)	75.39 (16.54)	44.64 (28.95)	62.90 (31.54)	62.10 (31.04)	56.59 (10.27)
PP^b^	19.10 (16.39)	51.43 (22.59)	57.02 (25.80)	52.63 (21.18)	75.44 (23.28)	60.09 (23.50)	54.17 (36.49)	52.78 (23.70)	69.85 (37.00)	54.98 (16.88)
SP^b^	25.61 (17.06)	51.76 (26.07)	65.20 (23.96)	57.75 (23.88)	73.03 (23.94)	69.52 (19.22)	48.37 (32.12)	51.56 (30.25)	67.55 (35.20)	56.56 (12.77)
CIS^b^	50.52 (26.62)	59.72 (26.41)	63.89 (15.51)	67.71 (14.48)	86.11 (13.61)	66.67 (15.81)	70.83 (31.46)	58.33 (28.14)	62.50 (26.22)	64.77 (8.95)
p**	**0.001**	0.82	0.61	0.14	0.29	0.13	0.45	0.30	0.63	0.73

Cognitive function*										

No deficit	31.03 (20.73)	52.08 (25.34)	61.42 (22.04)	58.22 (23.30)	74.27 (22.55)	67.26 (18.25)	52.55 (29.53)	59.72 (27.00)	63.39 (30.97)	57.10 (12.16)
Deficit	28.73 (22.53)	50.71 (24.13)	60.66 (26.65)	52.14 (26.00)	67.98 (28.81)	70.72 (20.45)	44.20 (36.08)	50.00 (32.57)	72.26 (36.44)	54.82 (14.14)
p*	0.34	0.86	1.00	0.25	0.42	0.36	0.31	0.15	0.11	0.68

EDSS^d^***	-0.40	0.09	0.15	0.12	0.08	-0.12	0.10	-0.06	0.17	0.12
p	**0.001**	0.37	0.13	0.23	0.43	0.24	0.37	0.58	0.09	0.30

Disease duration***	-0.05	-0.07	0.08	0.03	-0.13	-0.04	0.05	0.03	0.10	0.06
p	0.59	0.48	0.45	0.76	0.16	0.68	0.64	0.80	0.32	0.56

Age***	-0.05	0.13	0.07	0.06	-0.07	0.06	-0.04	0.18	0.13	0.06
p	0.57	0.17	0.47	0.54	0.46	0.55	0.71	0.07	0.19	0.59

^a^ADL activity of daily living, PWB psychological well-being, RFr relationships with friends, SPT symptoms, RFa relationships with family, RHCS relationships with health care system, SSL sentimental and sexual life, COP coping, REJ rejection

^b^RR Relapsing remitting, PP Primary progressive, SP Secondary progressive, CIS Clinically isolated syndrome

^c^Cognitive function is defined as a deficit from BRB-N with at least three of eight impaired subtests

^d^EDSS Expanded Disability Status Scale

* mean (standard deviation), p: p-value Mann-Whitney test

** mean (standard deviation), p: p-value Kruskal-Wallis test

*** Spearman's correlation coefficient, p: p-value Spearman's test

Bold values: p < 0.05

**Table 4:** Predictive factors for MusiQoL dimensions and index: multivariate analysis (standardised beta coefficient)

		ADL^a^	PWB^a^	RFr^a^	SPT^a^	RFa^a^	RHCS^a^	SSL^a^	COP^a^	REJ^a^	Index
Gender (0 women, 1 men)	β*	-0.011	0.161	**-0.563**	-0.017	-0.036	-0.302	**-0.416**	0.329	0.285	0.026
	p*	0.938	0.314	**0.004**	0.922	0.837	0.052	**0.037**	0.057	0.158	0.886

Marital status (0 single, 1 couple)	β*	-0.077	-0.015	**0.421**	0.075	**0.459**	**0.722**	0.261	-0.011	-0.013	**0.526**
	p*	0.571	0.919	**0.02**	0.642	**0.008**	**<10-3**	0.130	0.944	0.945	**0.007**

CIS^b ^(0 PP^b^)	β*	-0.312	**-0.461**	-0.106	-0.226	-0.087	**-0.461**	0.070	-0.345	-0.284	-0.309
	p*	0.092	**0.027**	0.644	0.297	0.690	**0.019**	0.738	0.107	0.250	0.124

SP^b ^(0 PP^b^)	β*	-0.013	-0.049	0.392	-0.158	0.182	-0.130	0.416	-0.112	0.135	0.127
	p*	0.946	0.821	0.138	0.507	0.449	0.534	0.177	0.631	0.620	0.670

RR^b ^(0 PP^b^)	β*	-0.389	-0.415	-0.037	-0.369	0.025	**-0.654**	0.285	**-0.500**	-0.271	-0.418
	p*	0.067	0.077	0.890	0.140	0.922	**0.004**	0.317	**0.043**	0.325	0.117

Age	β*	0.159	0.285	-0.368	**0.363**	-0.171	-0.045	-0.295	**0.357**	-0.004	-0.260
	p*	0.246	0.064	0.056	**0.030**	0.299	0.750	0.105	**0.029**	0.980	0.132

EDSS^c^	β*	**-0.421**	**0.429**	0.214	0.171	0.274	**0.575**	0.401	0.376	0.332	**0.633**
	p*	**0.027**	**0.038**	0.360	0.436	0.214	**0.005**	0.084	0.085	0.190	**0.006**

Disease duration	β*	**0.361**	-0.021	0.135	-0.071	-0.203	-0.360	-0.185	0.085	0.295	-0.182
	p*	**0.044**	0.908	0.565	0.731	0.338	0.054	0.449	0.674	0.209	0.434

MFIS-To^d^	β*	**-0.324**	-0.272	0.187	**-0.680**	0.113	**0.373**	-0.085	-0.023	-0.061	0.130
	p*	**0.043**	0.117	0.353	**0.001**	0.550	**0.027**	0.670	0.896	0.770	0.493

BDI^e^	β*	-0.142	**-0.317**	-0.115	0.318	-0.317	-0.116	-0.061	**-0.343**	-0.200	**-0.413**
	p*	0.323	**0.046**	0.530	0.068	0.072	0.438	0.728	**0.044**	0.304	**0.018**

SRT-L^f^	β*	-0.259	0.155	-0.433	-0.071	-0.194	-0.140	-0.456	0.071	0.202	-0.228
	p*	0.152	0.431	0.069	0.739	0.343	0.452	0.068	0.732	0.401	0.276

SRT-D^f^	β*	0.121	-0.075	-0.171	0.181	-0.009	-0.210	0.155	0.133	-0.208	0.035
	p*	0.482	0.692	0.439	0.378	0.965	0.246	0.518	0.507	0.366	0.864

SPART-T^f^	β*	0.014	0.184	-0.086	0.034	-0.005	**-0.429**	0.004	0.307	0.120	-0.152
	p*	0.928	0.270	0.667	0.852	0.977	**0.010**	0.983	0.087	0.552	0.413

WLG^f^	β*	0.244	0.072	0.196	0.220	-0.167	-0.100	0.392	-0.042	-0.023	0.078
	p*	0.084	0.640	0.301	0.186	0.312	0.489	0.056	0.795	0.902	0.666

^a^ADL activity of daily living, PWB psychological well-being, RFr relationships with friends, SPT symptoms, RFa relationships with family, RHCS relationships with health care system, SSL sentimental and sexual life, COP coping, REJ rejection

^b^RR Relapsing remitting, PP Primary progressive, SP Secondary progressive, CIS Clinically isolated syndrome

^c^EDSS Expanded Disability Status Scale

^d^MFIS-To Modified Fatigue Impact Scale total

^e^BDI Beck Depression Inventory

^f^SRT-L Selective Reminding Test Long-term, SRT-D Selective Reminding Test Delayed, SPART-T SPAtial Recall Test Total, WLG Word List Generation test

*β: standardised beta coefficient (β represents the change of the standard deviation in QoL score resulting from a change of one standard deviation in the independent variable); p: p-value

Bold values: p < 0.05

## Discussion

In the last few decades, there has been considerable expansion of the literature that addresses cognitive issues in MS [25]. While the nature of this cognitive dysfunction is relatively well described, prior studies of the relationship between cognitive impairment and QoL have been contradictory. To our knowledge, only one recent study has described these links using both a standardised cognitive evaluation (BRB-N) and a specific self-reported QoL questionnaire [16]. This work enrolled only patients with early MS, and depression was the only controlled parameter. Our study proposed a similar study design but included all MS subtypes and considered more key confounding factors. Consistent with Glanz's study [16], QoL was weakly or negligibly related to cognitive function. The link of interest (connection between visuospatial learning and the relationship with health care systems) has not been described elsewhere. This link did not seem substantial because no robust clinical hypothesis supported or denied this association.

Lovera and colleagues did not find relationships between attention or memory impairment and scores on the Perceived Deficits Questionnaire, which is restricted to assessment of self-perceived cognitive difficulties [11]. Rao found only non-significant trends between cognitive impairment and QoL as assessed by a generic questionnaire, the Sickness Impact Profile [10]. In Montel's study, a negative impact of cognitive impairment (defined by frontal lobe dysfunction) was identified only in the context of the mental health limitations domain of the SEP-59 questionnaire [12]. Mental and physical health composites of the MSQOL-54 were not predicted by cognitive functions in Benedict's report [7]. Cognitive status was not identified as a QoL predictive factor by Amato et al. [13].

Other authors have reported a more obvious association between cognitive deficits and self-reported outcomes, including QoL. Gold and colleagues identified lower QoL levels with the MS-specific Hamburg Quality of Life Questionnaire in cognitively impaired patients relative to cognitively preserved patients, but cognitive impairment was defined with a single attention-memory test (SDMT) [15]. In Miller's study, correlations between QoL (using generic questionnaires) and the Multiple Sclerosis Functional Composite were described, but the latter is not a strictly cognitive assessment tool but instead includes two clinical dimensions [14]. Because the MusiQoL is based on the concept of a health-related quality of life measure, no strong correlations with cognitive status are expected or sought. This result reinforces the validity and the acceptability of this questionnaire and is consistent with studies reporting that cognitively disabled patients are able to give consistent answers to questionnaires [15]. Finally, Benito-Leon's study reported associations between lower cognitive scores (MMSE) and lower QoL levels (Functional Assessment of MS) [5].

The existing data regarding factors that predict QoL in MS cases is somewhat contradictory. We found that gender was not linked to the global QoL score, as reported elsewhere [5,7,22,26]; other authors have shown that women with MS reported poorer QoL than men [27]. On the other hand, women reported worse QoL than men for dimensions such as coping and rejection, suggesting that women may be more vulnerable, and a higher QoL for relationships with friends and sentimental life. Similarly, subjects in a relationship described a higher QoL than single individuals in the MusiQoL index and in three dimensions (friends, family and health care relationships), though this association is not always found [28].

The disability level influenced a subset of QoL aspects, but the direction of this effect varied. Consistent with previous works, high disability was related to poorer QoL related to activities of daily living. Patients with higher disability, defined either by EDSS [6,7] or by other clinical rating scales [29], revealed significantly worse QoL than those with less pronounced disabilities. Conversely, severe disability was associated with better global QoL scores and a higher score in the psychological well-being and health care relationships dimensions, suggesting the potential relevance of coping strategies [30]. These weak and contradictory relationships between disability and QoL [8] confirm that clinical assessment does not reflect all the aspects that patients consider important in their life. We are reminded of the need to use a multi-dimensional approach for QoL assessment [31]. Disease duration was poorly associated with some QoL aspects, as already described [32]. Age is not related to QoL, but the literature reports contradictory results with either poorer [33] or better QoL [34] in older MS subjects. Finally, the strong influence of depression and fatigue as independent predictors of some aspects of QoL in MS patients was confirmed [8,11,35]. These parameters may be of significant clinical value for health care workers. Indeed, they can detect, prevent, or manage depression or fatigue possibly impacting QoL for MS patients. Our small sample size did not allow us to identify other factors predictive of QoL that have been reported elsewhere. We were unable to confirm the impact of anxiety [36] because this data was not collected in our study.

We note several strengths and limitations of this study:

1) The sample size was arguably too small. When we tried to identify linked factors using the multivariate approach, moderate associations may have been missed due to low statistical power. Nevertheless, the literature includes several studies with similar or smaller sample sizes [7,11,12].

2) The representativeness of our sample should be questioned. Compared to the international and European MS populations [22,37], our patients had a higher sex-ratio (0.41 and 0.60, respectively), a more severe disability profile (EDSS median 3.2 and 4.1, respectively), and a higher proportion of secondary progressive MS (21% and 36%, respectively). These disparities may partially explain the lower QoL scores reported by this population compared to others.

3) We are concerned about our neurocognitive assessment approach using BRB-N. Two neuropsychological batteries have been well-validated. The BRB-N and the MACFIMS [38] have comparable sensitivity among MS patients [39]. We chose to use the BRB-N because it was the most widely used at the beginning of this study. However, the BRB-N has several notable disadvantages: i) most studies have used a limited number of subtests from the complete set, and these truncated results are not readily comparable with studies that use the complete battery; ii) the executive function evaluation is not satisfactory; iii) the test performance requires fine visual acuity or motor speed.

4) There is no consensus on the definition of cognitive impairment, as underlined by Achiron and Barak [40]. Many studies define the cognitive impairment of a MS sample in comparison to a control group, while other studies use available normative values. Our choice of European norms [24], defined from a sample that included European populations, is questionable. Alternative normative values have been defined in Dutch [41], Italian [42], and Spanish [43] populations. French norms were proposed by Dujardin et al. [44], but their version differed from the standard BRB-N in terms of the content and the number of subtests. Furthermore, a patient may be considered cognitively impaired for one test if the score is less than 2 SDs [45,46], less than 1.5 SDs [47,48], or less than the fifth percentile [49] of healthy controls. A patient can be considered cognitively impaired for a global battery in the case of at least three impaired tests [24,46,50], or two impaired tests [49], or even just one impaired test [45]. The proportion of cognitively impaired patients depended on these definitions. These disparities make comparisons difficult between studies.

5) We did not assess thoroughly cognitive QoL nor specific cognitive dysfunction on daily functioning. Further studies should try to disentangle the impact of different cognitive domains on cognitive QoL on one hand and on cognitive daily functioning on the other hand.

## Conclusion

Although cognitive impairment is an important symptom in MS, its relationship with QoL appears to be contradictory. The present study, using a standardised neuropsychological battery and a disease-specific patient-based instrument, demonstrated the weak and scarce associations between cognitive disturbances and QoL alterations when confounding factors were accounted for. These preliminary results need to be confirmed with larger samples using more accurate tests of cognitive function.

## Competing interests

The authors declare that they have no competing interests.

## Authors' contributions

Conception and design: PA, JP, MCS. Study coordination: PA, JP, KBB. Inclusion and clinical data collection: IK, JP. Acquisition of cognitive data: FR, VA. Analysis of data: KBB. Interpretation of data: KBB, PA, JP, FR, VA. Drafting and writing of manuscript: KBB, PA. Revision of manuscript: PA, JP, FR, MCS, VA, IK.

All authors read and approved the final manuscript.

## Pre-publication history

The pre-publication history for this paper can be accessed here:

http://www.biomedcentral.com/1471-2377/11/17/prepub
